# Antioxidant and Antiproliferative Activities of *Eclipta prostrata* (L.) L. Extract and Isolated Compounds

**DOI:** 10.3390/molecules28217354

**Published:** 2023-10-30

**Authors:** Jinfeng Yang, Joo Seok Kim, Yong Soo Kwon, Eun Soo Seong, Myong Jo Kim

**Affiliations:** 1 Research Institute of Food Science & Engineering Technology, Hezhou University, Hezhou 542899, China; yjf19830224@126.com; 2 Bioherb Research Institute, Kangwon National University, Chuncheon 200-701, Republic of Korea; 3 Department of Pharmacy, Kangwon National University, Chuncheon 200-701, Republic of Korea

**Keywords:** *Eclipta prostrata* (L.) L., antioxidant activity, antiproliferative, flavonoids

## Abstract

The primary objective of this study was to elucidate the chemical composition, antioxidant properties, and antiproliferative activities of *Eclipta prostrata* extracts. Two flavonoids, 3′-O-methylorobol and apigenin 7-sulfate, were isolated from the ethyl acetate (EtOAc) extract of *E. prostrata*. The total phenolic and flavonoid contents of the *E. prostrata* extracts, as well as their overall antioxidant activities as measured using the 2,2-diphenyl-1-picrylhydrazyl and reducing power assays, were investigated. The *E. prostrata* EtOAc extract exhibited significantly greater antioxidant activities in both assays and higher phenol and flavonoid contents than the other extracts. The potential antiproliferative properties of the *E. prostrata* extracts and isolated compounds were investigated in vitro against the AGS, A549, and HT-29 cancer cell lines and the normal human HEK-293 cell line using the MTT assay. Annexin V-FITC/PI staining analysis and quantitative real-time PCR were used to assess AGS cell apoptosis. At a concentration of 100 µg/mL, the EtOAc extract of *E. prostrata* reduced AGS cell viability and proliferation by inducing apoptosis through the alteration of gene expression in the apoptotic cascade. These results highlight *E. prostrata* as a promising source of anticancer compounds.

## 1. Introduction

*Eclipta prostrata* (L.) L. is an annual herb that thrives abundantly in tropical, subtropical, and warm temperate regions across the globe. *E. prostrata* is a medium-sized, branching plant that ranges from 20 to 90 cm in height [1]. In the Traditional Chinese Medicine (TCM) system, *E. prostrata* is classified as a yin-nourishing herb and has a long history of use for alleviating conditions such as loose teeth, premature graying of hair, dizziness, tinnitus, and hemorrhage [2]. TCM often combines leaf juice with honey to treat catarrh in infants, whereas a mixture of shoot juice and mustard oil is administered for diarrhea and dysentery. The entire plant is utilized to address symptoms associated with hepatitis, itching, hemoptysis, bleeding, hematuria, diarrhea, and diphtheria [1]. In Nepal, the leaves and shoots of *E. prostrata* are used to prevent infections in wounds and facilitate wound healing [3]. Furthermore, various ethnic groups in certain South American regions have adopted *E. prostrata* as a remedy for snakebites [4].

Some studies have investigated the chemical compounds of *E. prostrata*, including flavonoids, phenolics, proteins, alkaloids, lipids, and phytosterols [5]. Flavonoids, members of the polyphenol superfamily, are synthesized by plants and play a role in the regulation of plant growth and development [6]. They are widely distributed throughout plant stems, leaves, blossoms, and fruits, and typically manifest as plant pigments in shades of yellow, light yellow, or even white. Natural flavonoids found in plants exhibit potent antioxidant properties and offer a plethora of other health benefits. Numerous studies have documented the diverse biological and pharmacological activities associated with flavonoids, including their anticancer, anti-inflammatory, and anti-amoebic properties [7,8,9]. As a result, flavonoids have gradually emerged as a focal point of research in disciplines such as biology, food science, and medicine. Kim et al. [10] reported that *E. prostrata* contains substantial quantities of flavonoid compounds with a distinct ortho-diphenolic structure, which makes them highly oxidizable.

Oxidative stress arises from an imbalance between the body’s antioxidant defense system and the generation of reactive oxygen species [11], which are produced through oxidation reactions within organisms [12]. The biochemical imbalance induced by reactive oxygen species can damage cell membranes, RNA, lipids, proteins, and DNA, which may harm cells, lead to cell death, and exacerbate various age-related chronic illnesses, including multiple sclerosis, Alzheimer’s disease, and cancer [6,13]. Antioxidants are therefore recognized as vital nutraceuticals. However, many synthetic antioxidants commonly used in the food industry as potential inhibitors of lipid peroxidation, such as butylated hydroxyanisole and butylated hydroxytoluene, can accumulate within the body, potentially causing liver damage and promoting carcinogenesis [14]. Owing to the increasing risk factors associated with various life-threatening diseases, there is a worldwide inclination to leverage natural antioxidants present in medicinal and dietary plants. Research indicates a negative correlation between the intake of antioxidant-rich foods, encompassing medicinal plants, and the occurrence of human diseases. Introducing natural antioxidants to the food, cosmetic, and therapeutic sectors provides a promising substitute for synthetic counterparts, boasting benefits like cost-effectiveness, seamless integration with dietary practices, and absence of adverse effects on the human body. Many antioxidant compounds, inherent to plant origins, have been recognized as scavengers of free radicals or active oxygen. Natural compounds derived from medicinal plants have ushered in a new era of safeguarding the human body from free radicals and slowing the progression of several chronic diseases [15].

Natural products are invaluable reservoirs for the treatment of a wide spectrum of human ailments. Remarkably, more than 60% of the presently employed anticancer agents originated in the natural world among plants, marine organisms, and microorganisms [16]. Epidemiological studies have indicated that antioxidant supplements may decrease the likelihood of cancer recurrence [17]. Furthermore, the consumption of flavone-rich foods and beverages has been linked to a reduced occurrence of cancer [18]. Experimental inquiries have revealed that methanolic extract derived from the pericarp of *Garcinia mangostana* exhibits notable antioxidant and anticancer potential across a range of bioassay systems and animal models, with direct relevance to human health conditions [19,20].

This study explored a solvent-based approach for screening and purifying the active compounds present in *E. prostrata* and assessed the antioxidant activities of these extracts. We further investigated the inhibitory effects of these extracts on the in vitro growth of human cancer cells and sought to elucidate the mechanisms underlying their antiproliferative activity.

## 2. Results and Discussion

### 2.1. Antioxidant Activities of E. prostrata Extracts

**Figure 1:**
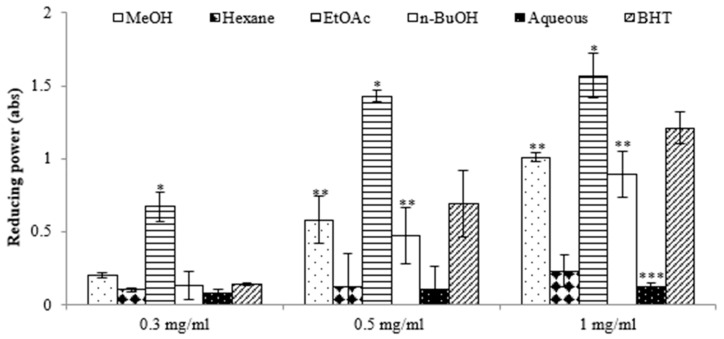
Reductive potential of different extracts from the methanol extract of *E. prostrate* by different solvents at various concentrations. (* *p* < 0.05, ** *p* < 0.01, *** *p* < 0.001).

The antioxidant potential of the extracts derived from *E. prostrata* was assessed using the 2,2-diphenyl-1-picrylhydrazyl (DPPH) assay. The results in Table 1 show that the ethyl acetate (EtOAc) extract exhibited a superior capacity to reduce DPPH radicals (IC_50_, 26.12 ± 1.83 μg/mL) compared with the other extracts, surpassing even that of the positive control, 2,6-di-tert-butyl-4-methylphenol (IC_50_, 109.61 ± 2.98 µg/mL). We also assessed the antioxidant potential of various extracts using the reducing power assay, which quantifies a broad spectrum of antioxidant activities and capacities to counteract the oxidative effects of reactive oxygen species over a wide range of concentrations [16]. The results revealed significant differences in reducing power among the extracts, and this power increased with rising concentrations. The highest reducing activity was observed in the EtOAc extract, with absorbance values of 0.67 at 0.3 mg/mL, 1.43 at 0.5 mg/mL, and 1.57 at 1 mg/mL (Figure 1). Previous reports have suggested that such reducing properties exert antioxidant action by donating a hydrogen atom to break the free radical chain. Increasing absorbance at 700 nm indicates an increase in reducing ability. The antioxidants present in the EtOAc extract of *E. prostrata* caused the reduction of Fe^3+^/ferricyanide complex to its ferrous form and, thus, proved the reducing power. Furthermore, we analyzed the total phenolic content (TPC) and total flavonoid content (TFC) of various extracts. As indicated in Table 1, the EtOAc extract exhibited the highest TPC and TFC (124.56 ± 1.35 mg GAE/g and 24.69 ± 0.48 mg QE/g, respectively), surpassing all other fractions. Conversely, the aqueous extract demonstrated the lowest TPC and TFC values.

Free radicals are involved in numerous pathological processes. Antioxidants neutralize free radicals, thus protecting us from a variety of diseases. They are effective either by scavenging reactive oxygen species or by enhancing antioxidant defense mechanisms. The electron-donation capacity of natural compounds can be assessed by decolorizing a purple solution of 2,2′-diphenyl-1-picrylhydrazyl (DPPH). This assay relies on scavenging DPPH by adding either a radical species or an antioxidant, leading to the decolorization of the DPPH solution. The degree of color change corresponds to the concentration and potency of antioxidants. A significant decrease in the absorbance of the reaction mixture indicates strong free radical scavenging activity of the evaluated compound [21]. Among the examined extracts in this study, ethyl acetate showed a significantly higher inhibition percentage and the highest total phenolic content. These findings suggest that the plant extract contains phytochemical constituents capable of donating hydrogen to free radicals, thereby scavenging potential damage.

Phenolic compounds, numbering over 10,000, form the primary class of phytochemicals. Furthermore, these phenolic compounds exhibit health-promoting effects, such as inhibiting platelet aggregation, exerting antitumor activity, defending against pathogens, and providing protection against the harmful effects of ultraviolet radiation. Phenolic compounds primarily function as antioxidants, defending against oxidative stress caused by reactive oxygen species (ROS) and free radicals in the human body [22].

Flavonoids, naturally occurring in plants, are believed to have positive effects on human health. Studies on flavonoid derivatives have uncovered a diverse range of activities, encompassing antibacterial, antiviral, anti-inflammatory, anticancer, and anti-allergic properties. Flavonoids have shown remarkable effectiveness as scavengers for a broad range of oxidizing molecules, including singlet oxygen and various free radicals [23], which are implicated in various diseases. This is consistent with similar findings in the literature regarding extracts from other plant products [24]. Our findings suggest that phenolics and flavonoids may be the main contributors to antioxidant activities, considering the observed IC50 values for the radical scavenging activity of ethyl acetate. Therefore, *E. prostrata* holds promise as a source of food antioxidants.

### 2.2. E. prostrata Affects Cancer Cell Growth

**Figure 2:**
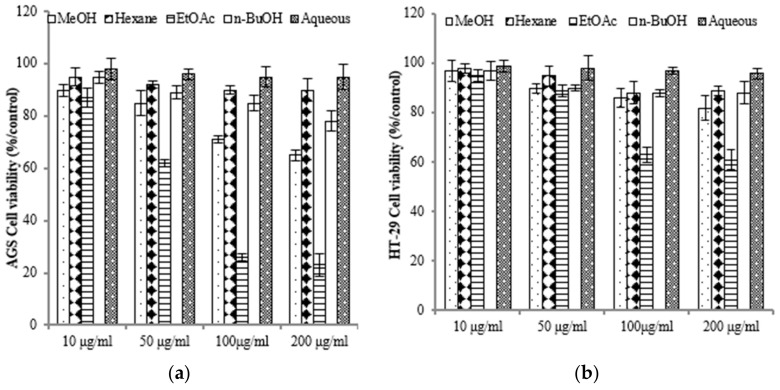

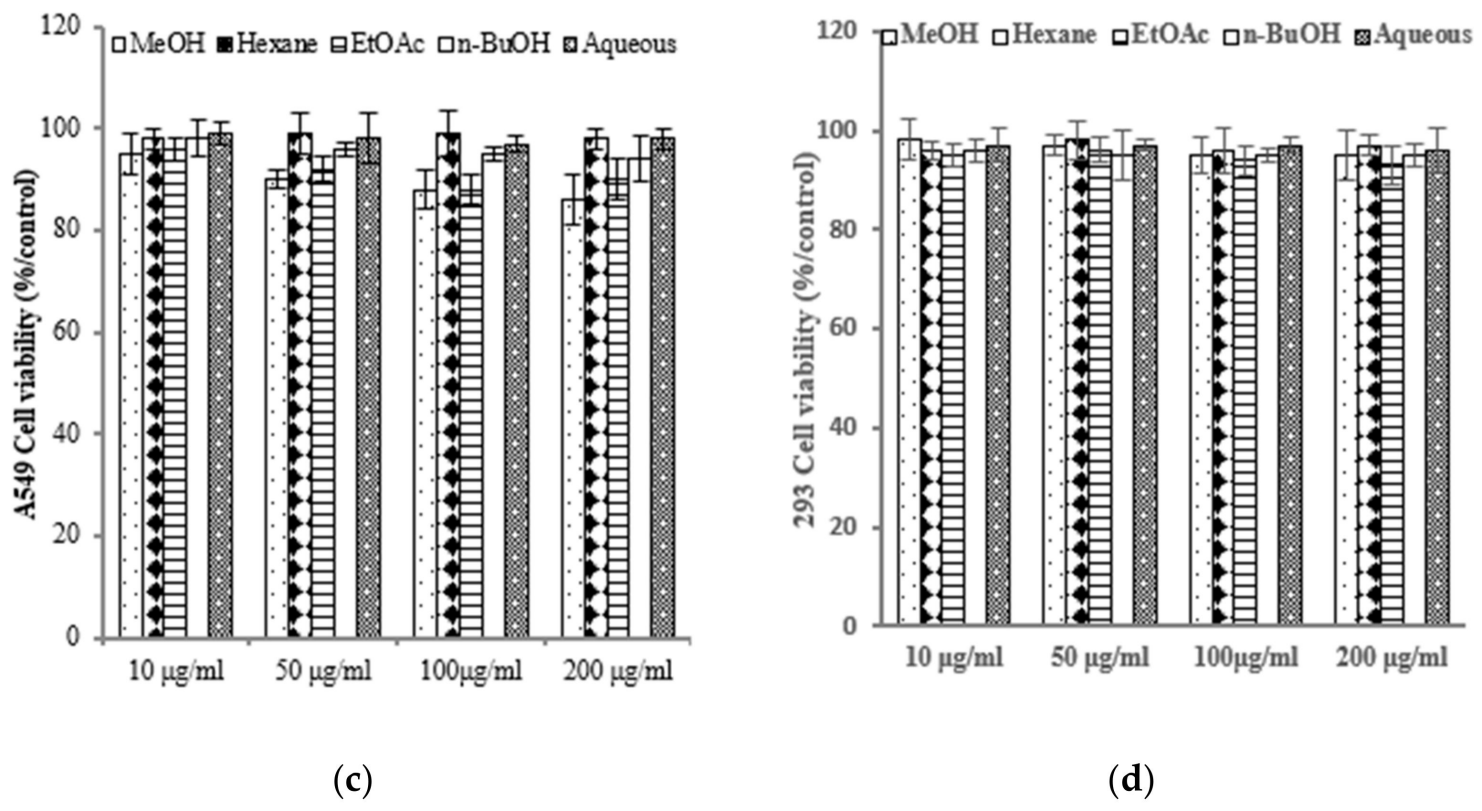
The anti-proliferative effect of *E. prostrate* extracts on human cancer cell lines. The indicated cell lines were treated with different extract dose, and cell viability was determined using a MTT assay. (**a**) human gastric carcinoma cells (AGS). (**b**) human colorectal carcinoma cells (HT-29). (**c**) human lung cancer cells (A549). (**d**) human embryonic kidney cells (293).

To assess the varying inhibitory potential of *E. prostrata* extracts on cancer cell proliferation, different concentrations of the extracts were used to treat cancer cells. The proliferation inhibition rate was then measured to identify the cancer cell lines most sensitive to *E. prostrata*. Figure 2 illustrates the impact of varying concentrations of *E. prostrata* on AGS, HT-29, and A549 cancer cells over 24 h. Cellular proliferation was consistently suppressed in all three types of cancer cells, and the inhibition rate steadily increased as the extract concentrations increased. Among the cancer cell lines tested, AGS cells were the most sensitive to the EtOAc extract, with cell viability decreasing in a dose-dependent manner (Figure 2a). The EtOAc extract with a concentration of 100 μg/mL induced approximately 70% cell death. No significant difference in cell viability was observed in EtOAc extract-treated normal HEK-293 cells (Figure 2d).

**Figure 3:**
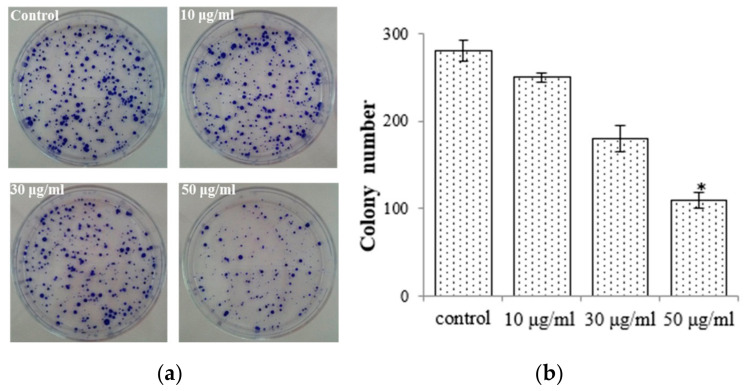
*E. prostrate* inhibits colony formation in AGS cancer cell colonies. (**a**) Colonies stained with crystal violet. (**b**) The number of colonies is significantly lower in cells treated with the EtOAc extract than in the control group (* *p* < 0.05).

To elucidate the antiproliferative impact of the EtOAc extract, we conducted colony formation assays with AGS cells while subjecting them to treatment with the EtOAc extract. We observed that colony formation was lower in the EtOAc extract-treated group (50 μg/mL: 108.7 ± 9.12) compared with the control group (275.6 ± 12.8; Figure 3). We also observed that the EtOAc extract exerted a substantial impact on the growth rate of AGS cells with a clear dose-dependent relationship (Figure 2a). These findings were corroborated by the results obtained from the colony formation assays. Consequently, *E. prostrata* could be considered a promising inhibitor of gastric carcinoma cell proliferation.

The experiments revealed high levels of phenolic and flavonoid contents in the EtOAc extract. These compounds were likely responsible for the observed strong anticancer activities. Many studies have identified polyphenolic compounds, particularly flavonoids, as widely recognized dietary antioxidants. These compounds are acknowledged for their potential anticancer properties. To date, extensive research has explored their capacity to impede the cell cycle, induce apoptotic cell death in diverse cancer cell lines, disrupt mitotic spindle formation, or inhibit angiogenesis. Thus, it is plausible that the growth inhibitory effect of the extract can be attributed, at least in part, to these phenolic compounds.

### 2.3. E. prostrata Induces Apoptosis of Gastric Carcinoma Cells

**Figure 4:**
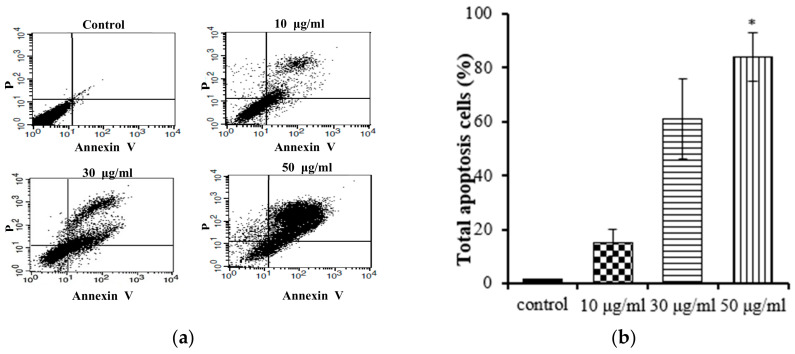
Flow cytometry results of EtOAc extract from *E. prostrate* induced AGS cell apoptosis: (**a**) dot plots generated via flow cytometry, depicting Annexin V-FITC/PI dual staining for the discernment of apoptosis; (**b**) flow cytometry results: AGS cells were incubated with EtOAc extract at 10, 30, and 50 μg/mL for 24 h (* *p* < 0.05).

Apoptosis and necrosis are two main types of cell death, and they play distinct roles in the context of cancer cells. Apoptosis, a cellular self-termination mechanism, is essential for eliminating tumors. This intricate process is characterized by membrane blebbing, cellular shrinkage, nuclear volume reduction, chromatin condensation, DNA fragmentation, and the formation of apoptotic bodies. Apoptosis can be initiated through a multitude of pathways originating within or outside of cells. Necrosis causes damage to cells and tissues and releases cytotoxins, which can further damage surrounding tissues. A number of studies have shown that inducing apoptosis in cancer cells can be an effective way to treat cancer [25]. We conducted further studies to investigate whether the EtOAc extract of *E. prostrata* could influence gastric carcinoma cell proliferation by inducing apoptosis. Annexin V-FITC/PI staining was used with flow cytometry to quantify the proportion of apoptotic cells in the total cell population. The Annexin V-FITC apoptosis detection kit is an invaluable tool for researchers studying apoptosis. This kit facilitates the monitoring of apoptosis progression in a cell population, the identification of cells undergoing apoptosis, and the quantification of apoptotic cells in a sample. As shown in Figure 4, the EtOAc extract (at concentrations of 10, 30, and 50 μg/mL) induced apoptosis in AGS cells; the apoptotic percentages ranged from 15% to 84% and followed a notable concentration-dependent increasing trend. These findings support the hypothesis that the EtOAc extract inhibits the proliferation of AGS cells by promoting apoptosis.

**Figure 5:**
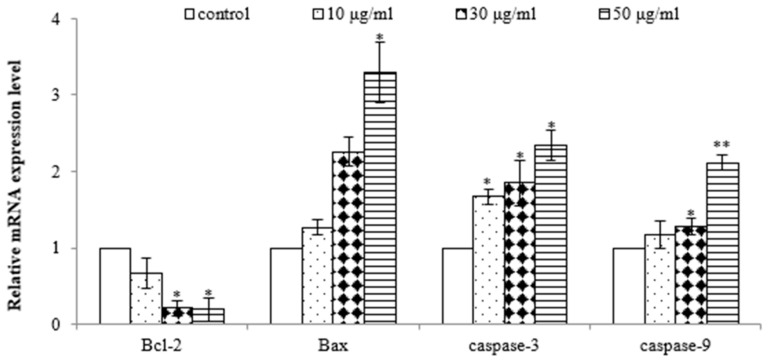
Effect of EtOAc extract from *E. prostrate* on expression of cell apoptosis genes in AGS cells (* *p* < 0.05, ** *p* < 0.01).

To further demonstrate that the EtOAc extract induced apoptosis in AGS cells, quantitative real-time PCR analysis was performed to assess the gene expression of Bax, Bcl-2, caspase-3, and caspase-9. As shown in Figure 5, the EtOAc extract induced dose-dependent upregulation of the gene expressions of Bax, caspase-3, and caspase-9 and downregulation of the gene expression of Bcl-2 in AGS cells. Bcl-2 and Bax play pivotal roles in the mitochondrial pathway of apoptosis [26]. Decreased Bcl-2 and increased Bax levels are necessary for the formation of pores in the mitochondrial membrane and, subsequently, the release of cytochrome c. Elevated levels of cytochrome c promptly trigger the activation of caspase-9 [27], a pro-apoptotic protein, which initiates the activation of its downstream effector, caspase-3, culminating in programmed cell demise [28]. Therefore, these results further demonstrate the potent antiproliferative and pro-apoptotic properties of the EtOAc extract of *E. prostrata* in SW620 cells, mediated by the downregulation of Bcl-2 and the upregulation of Bax, caspase-3, and caspase-9.

### 2.4. Isolation of Active Compounds

**Figure 6:**
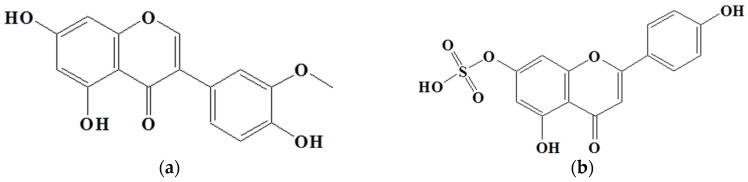
Structure of compounds from *E. prostrate*: (**a**) compound **1**: 3′-*O*-methylorobol; (**b**) compound **2**: apigenin 7-sulfate.

**Figure 7:**
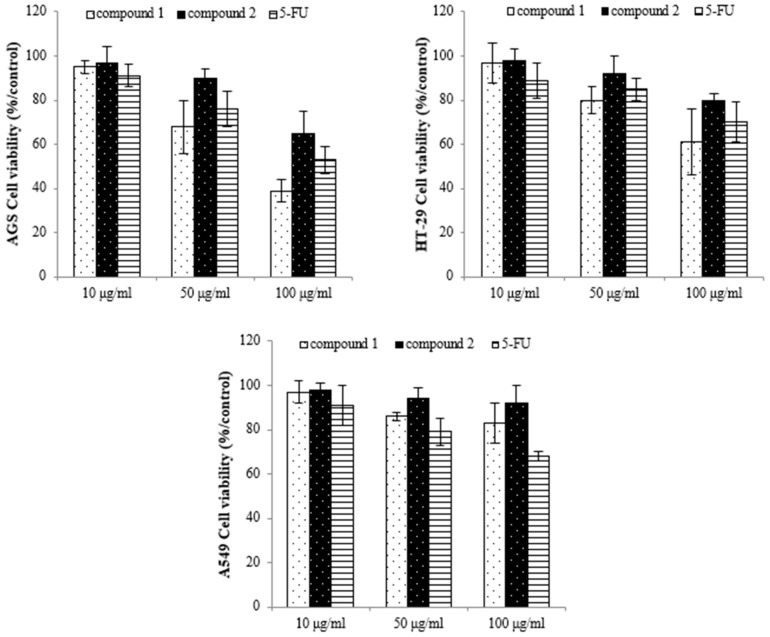
The inhibition effect of the compounds on cancer cell proliferation as assessed by using the MTT assay. Compound **1**: 3′-*O*-methylorobol; compound **2**: apigenin 7-sulfate; 5-FU: fluorouracil was used as the positive control.

To identify the active compounds responsible for inhibiting proliferation, we used column and thin-layer chromatography to isolate and quantify the active compounds in the EtOAc extract, resulting in the isolation of two pure flavonoids. The structures of compounds **1** and **2** are depicted in Figure 6. We identified compound **1** as a white powder material. The ^1^H-NMR spectrum shows signals at δ 6.2, 6.3, 6.8, 6.9, and 7.1 ppm. The signals at δ 6.2 and 6.3 ppm are single signals, and the δ 6.8 and 7.1 ppm signals are doublet signals with values of *J* = 8.15 and 1.91, respectively. The δ 6.9 ppm signals are multiplet signals with values of *J* = 1.93 and 1.95. The ^13^C-NMR spectrum shows signals at δ 94.22, 99.51, 104.72, 113.80, 115.67, 122.16, 122.21, 122.79, 147.10, 147.68, 154.54, 158.07, 162.18, 165.19, and 180.51 ppm. By comparing their ^1^H and ^13^C NMR data with the existing literature, compound **1** was identified as 3′-*O*-methylorobol. Compound **2** is a yellow powder. The ^1^H-NMR spectrum shows signals at δ 6.6, 6.7, 6.9, 7.0, and 7.8 ppm. The signals at δ 6.6 and 6.7 ppm are singlets, and the signals at δ 6.9, 7.0, and 7.8 ppm are doublets with values of *J* = 2.17, 8.76, 2.11, and 8.86, respectively. The ^13^C-NMR spectrum shows signals at δ 100.588, 104.639, 105.100, 108.635, 116.752, 117.498, 123.474, 130.081, 158.772, 160.511, 162.944, 163.398, 167.405, and 184.719 ppm. By comparing these results with the literature, compound **2** was identified as apigenin 7-sulfate [29]. The antiproliferative properties of various concentrations of the isolated compounds (**1** and **2**) were assessed against A549, HT-29, and AGS cancer cell lines using the MTT assay. As shown in Figure 7, the isolated compounds exhibited varying degrees of inhibition of cancer cell proliferation in a dose-dependent manner. For example, compound 1 exhibited a remarkable 60% reduction in AGS cell proliferation at a concentration of 100 µg/mL. We subsequently evaluated the antiproliferative effects of the two compounds on human lung cancer (A549) and colorectal cancer (HT-29) cell lines. Compound **2** exhibited more pronounced antiproliferative activity against the colorectal cancer cell line compared to the lung cancer cell line.

3′-*O*-methylorobol, an antioxidant flavonoid, has been isolated from several medicinal plants, including *Millettia nitida var. hirsutissima*, *Flemingia philippinensis*, and *Dalbergia parviflora* [30]. Despite limited research on its bioactivity, 3′-*O*-methylorobol has been shown to exhibit moderate antioxidant activity in the DPPH free radical scavenging assay, potential analgesic properties, increased osteoblast differentiation, and anti-itch efficacy comparable to cyproheptadine in a histaminergic itch mouse model [31]. Apigenin 7-sulfate is an isoflavone that naturally occurs in certain species of plants. Though no antiproliferative effects against cancer have previously been reported for apigenin 7-sulfate, the present study demonstrated that both flavonoid compounds are potent cancer inhibitors. Flavonoids have been shown to inhibit the growth of a variety of human cancer cell lines, including DLA, MCF-7, A549, and HepG_2_, by triggering apoptosis, slowing cellular proliferation, and inhibiting metastasis by targeting various signaling pathways [7]. Our results further support that flavonoids may be used to inhibit cancer growth.

## 3. Materials and Methods

### 3.1. Plant Materials

The entire *E. prostrate* plant was provided by the Institute of Eco-environmental Science in Kangwon, Republic of Korea. The air-dried plant material (290 g) was subjected to three methanol extractions at room temperature, followed by concentration under reduced pressure at 50 °C until dryness. The resulting crude extract was suspended in water and subsequently partitioned into *n*-hexane, ethyl acetate (EtOAc), and butanol (BuOH) fractions. The EtOAc fraction was subjected to chromatography on silica gel using a mixture of trichloromethane and methanol (15:1–0:1) as eluents, resulting in the isolation of eight fractions (Fr.1-Fr.8). Fr.2 was further purified via silica gel column chromatography, utilizing a mixture of benzene and ethyl acetate (50:10) as eluents, which yielded four subfractions (Fr.2-1-Fr.2-4). Subfraction Fr.2-2 underwent purification via RP-18 column chromatography using a gradient elution with water and methanol (90:10-0:100), resulting in the isolation of compound **1** (413.7 mg). Fr.5 was subjected to RP-18 column chromatography with a gradient elution of water and methanol (60:40), yielding six fractions (Fr.5-1-Fr.5-6). Subfraction Fr.5-1 was further separated on a silica gel column using a mixture of methylene chloride, methanol, and water (10:4:0.5) as eluents, yielding four subfractions (Fr. 5-1-1-Fr. 5-1-4). Subfraction Fr.5-1-2 was subsequently purified via Sephadex LH-20 to obtain compound **2** (75.7 mg).

### 3.2. DPPH Free Radical Scavenging Activity

The antioxidant activity of the *E. prostrate* extracts was determined using the DPPH radical scavenging assay. The *E. prostrate* extracts were diluted with methanol to final concentrations of 10, 50, 100, and 500 µg/mL. DPPH solution was introduced to each sample, and the amalgamation was permitted to undergo reaction at ambient temperature for a duration of 30 min. The samples’ absorbance was determined at 517 nm [32].

### 3.3. Analysis of Reducing-Power Activity

Various concentrations of the extracts were homogenized with 0.5 mL of 0.2 M NaH_2_PO_4_ (pH 6.6) and 0.5 mL of 1% K_3_[Fe(CN)_6_]. The mixture was then incubated at 50 °C for 20 min, followed by the addition of 2.5 mL of 10% CCl_3_COOH. After centrifugation at 650 revolutions per minute for 10 min, the upper layer (0.5 mL) was combined with an equal volume of deionized water and 0.1 mL of 0.1% FeCl_3_. The absorbance was determined at a wavelength of 700 nm. Butylated hydroxytoluene (BHT) served as the reference standard [16].

### 3.4. Total Phenolic and Flavonoid Contents

Total phenolic content was measured at 725 nm using the Folin–Ciocalteau method [33]. In this procedure, each extract (100 μL) was combined with 0.05 mL of Folin phenol reagent and allowed to react for 3 min. Subsequently, 0.3 mL of a 20% Na_2_CO_3_ solution was added. After a 30 min incubation period, the absorbance of the reaction was measured. The total phenolic content was quantified in terms of gallic acid equivalents (GAE) per milligram of each extract. The total flavonoid content was determined using the method described by Park et al. Specifically, 0.2 mL of the extract was mixed with 0.1 mL of 10% Al(NO_3_)_3_, 0.1 mL of 1 M CH_3_COOK, and 4.6 mL of 80% ethanol. These mixtures were incubated at room temperature for 40 min, after which the absorbance was measured at 415 nm [34].

### 3.5. Cell Culture

The tumor cell lines, including human lung cancer cells (A549), human gastric carcinoma cells (AGS), human colorectal carcinoma cells (HT-29), and human embryonic kidney cells (293), were sourced from the Korean Cell Line Bank. A549, HT-29, and AGS cells were maintained in RPMI 1640 medium containing 10% fetal bovine serum (FBS) and 10 μg/mL of penicillin. A total of 293 cells were cultured in DMEM medium supplemented with 10% FBS and 10% penicillin. All cell lines were incubated at 37 °C and 5% CO_2_.

### 3.6. Cell Viability

Cell viability was assessed using the MTT assay. Cells (1 × 10^4^ well) were seeded in 96-well plates with 100 μL of medium per well. After overnight incubation, the cells were exposed to 100 μL of various concentrations (10, 50, 100, 200 μg/mL) of *E. prostrate* for a duration of 24 h. Subsequently, 100 μL of a 5 mg/mL MTT solution was added to each well and incubated for an additional 4 h. After that, the supernatant was carefully removed, and 100 μL of DMSO was added to each well, followed by shaking for 15 min. The absorbance at 570 nm was then measured using a microplate reader [35].

### 3.7. Colony Formation Assay

Tumor cells were dissociated using a trypsin solution during their logarithmic growth phase to create a single-cell suspension in the culture medium. Subsequently, a cell counting chamber was used to determine the cell count within a 10 μL single-cell suspension. This was performed using an inverted microscope. These cells were meticulously transferred into six-well culture plates, each well equipped with a sterile glass coverslip. An amount of 500–1000 cells was seeded into each well. The culture medium was replenished every 3 days until visible cell colonies could be observed without the aid of a microscope.

### 3.8. Cell Cycle Assay

Cells were seeded into 6-well plates and incubated for a duration of 24 h. Following this, the medium was substituted with fresh medium, and the cells were subjected to varying concentrations (10, 30, and 50 μg/mL) of the EtOAc extract for 24 h. The cells were then digested with 0.05% trypsin-EDTA for 3 min, collected, and centrifuged at 1200× *g* for 5 min. The cells underwent two washes with PBS and were subsequently reconstituted in 1× binding buffer at a concentration of 1 × 10^5^ cells/mL. All samples were kept on ice. Then, 5 μL of Annexin V-FITC and 5 μL of PI were introduced to each sample, and the cells underwent incubation for 10 min. The cells were then examined using FACS.

### 3.9. qRT-PCR Analysis

AGS cells were subjected to the intended concentrations of the samples. Subsequently, the total cellular RNA was isolated using TRIzol reagent (Invitrogen, Danvers, MA, USA) in accordance with the manufacturer’s instructions. The primer sequences can be found in Table 2. The quantitative analysis of the target gene was performed using the NanoHelix kit. The RT-qPCR program was configured as follows: an initial denaturation step at 95 °C for 30 s with a total volume of 15 µL, followed by 45 cycles of denaturation at 95 °C for 10 s, annealing at 55–60 °C for 35 s, and extension at 72 °C for 40 s. The relative expression level of the target gene was determined by the system, with β-actin serving as the internal reference gene.

### 3.10. Statistics

All experiments were performed in triplicate. The data were subjected to analysis using one-way ANOVA, and the results are presented as the mean and standard deviation. *p* < 0.05 was considered statistically significant.

## 4. Conclusions

In summary, the EtOAc extract of *E. prostrata* is rich in phenolic and flavonoid compounds and exhibits potent free radical scavenging activities and reducing power. Additionally, the extract induces apoptosis in stomach cancer cells by altering the expression of key genes in the apoptotic cascade. Two flavonoids, 3′-*O*-methylorobol and apigenin 7-sulfate, were isolated from *E. prostrata* and showed promising antiproliferative activity against AGS stomach cancer cells and HT-29 colon cancer cells in vitro. Our discoveries establish a scientific foundation for the longstanding utilization of *E. prostrata* in cancer management and endorse the use of this botanical specimen as a wellspring of novel anticancer agents and antioxidants.

## Figures and Tables

**Table 1:** Antioxidant capacity and total phenolic and total flavonoid contents of extracts from *E. prostrate*.

Extracts	DPPH Radical Scavenging Activity IC_50_ (μg/mL)	Total Phenolic Contents (mg GAE/g)	Total Flavonoid Contents (mg QE/g)
MeOH	88.53 ± 0.83 ^b^	63.22 ± 2.90 ^b^	15.62 ± 0.29 ^b^
Hexane	189.77 ± 5.77 ^c^	14.82 ± 2.09 ^d^	2.11 ± 0.01 ^d^
EtOAc	26.12 ± 1.83 ^a^	124.56 ± 1.35 ^a^	24.69 ± 0.48 ^a^
*n*-BuOH	396.67 ± 8.72 ^d^	37.39 ± 4.23 ^c^	5.22 ± 0.64 ^c^
Aqueous	ND	5.27 ± 0.22 ^e^	1.58 ± 0.25 ^d^
BHT	109.61 ± 2.98		

ND: not detected. ^a–e^ Values with different letter superscripts are significantly different at *p* < 0.05 as per Duncan’s multiple range test.

**Table 2:** Gene annotations and primer sequences used in RT-PCR analysis.

Primer	Sequences	
*Bax*	Forward	5′-GTTGTCGCCCTTTTCTACTTTG-3′
	Reverse	5′-GCACTTTCTTCGCAGTTTCC-3′
*Bcl-2*	Forward	5′-GTGGATGACTGAGTACCTGAAC-3′
	Reverse	5′-GGACATCAGTCGCTTCAGTG-3′
*Caspase-3*	Forward	5′-GGATGGGTGCTATTGTGAGG-3′
	Reverse	5′-TGGGATTTCAAGGCGACG-3′
*Caspase-9*	Forward	5′-AGAGATTCGCAAACCAGAGG-3′
	Reverse	5′-CACGGCAGAAGTTCACATTG-3′
*β-actin*	Forward	5′-ACCACACCTTCTACAATGAGC-3′
	Reverse	5′-GCGTACAGGGATAGCACAG-3′

## Data Availability

The data presented in this study are contained within the article.
